# Supplementary material for: Effect of active follow-up of women with previous cesarean delivery on uptake of timely safe obstetric and surgical care: Comparison between pre-intervention and intervention cohorts in Rwanda
Source: PLoS One. 2025 Jun 13;20(6):e0325884. doi: 10.1371/journal.pone.0325884 (PMC12165374; doi:10.1371/journal.pone.0325884)
Supplement: Attachment — Submitted filename: Response to Reviewers.docx [file pone.0325884.s002.docx]

**Response to the Editor and Reviewers' Comments**

PONE-D-24-21352

Effect of active follow-up of women with previous cesarean delivery on uptake of timely safe obstetric and surgical care: comparison between pre-intervention and intervention cohorts in Rwanda

**A. RESPONSE TO MAJOR ISSUES**

**Response to comment 1:**

- Thank you for your feedback regarding the clarity of the study's objective. We have revised the objective to ensure it accurately reflects the scope of the study and aligns with the analyses conducted. The updated objective is now twofold: **“**The objective of this study is twofold: (1) to evaluate the effect of the intervention on the emergency CD rate among women with previous cesarean delivery, and (2) to identify factors associated with emergency cesarean deliveries.” This revised objective addresses the concern about ambiguity in the study’s aim and is clearly stated in the manuscript (see lines 91–92). It clarifies the dual focus of the study and ensures consistency across the methods, and results sections.
- We appreciate your observation regarding the outcomes analyzed in the study. We would like to clarify that this study has a single outcome: the rate of emergency cesarean deliveries, as mentioned in line 101 of the revised manuscript. The text in lines 102–107 provides a detailed explanation of how the outcome of emergency cesarean delivery was defined. Specifically, we outlined two scenarios under which a cesarean delivery was classified as an emergency: a) a woman with a previous CD was admitted with contractions and a physician’s documented the need for emergency intervention, or b) a woman with a previous CD had a delivery plan and presented to the hospital on the scheduled date and was admitted without contractions; however, while awaiting the procedure, she began experiencing contractions and the physician confirmed the need for emergency intervention. To ensure further clarity and address potential ambiguity, we revised the manuscript to explicitly state that these criteria were applied consistently across both the pre-intervention and intervention cohorts to ensure uniformity in outcome classification. This addition is reflected in line 108-109.
- We appreciate your comment regarding the inclusion of low birth weight as a predictor for emergency cesarean delivery. Upon careful review, we recognize that including low birth weight as a predictor in the original manuscript was inappropriate, as it violates the temporal sequence between cause and effect. Specifically, birth weight is determined after the occurrence of the outcome (emergency cesarean delivery), which makes its use as a predictor in a cohort study design methodologically incorrect. In response to this concern, we have decided to remove low birth weight from the analysis.

**Response to comment 2:**

- We appreciate the reviewer’s observation regarding potential temporal confounding. During the study period, the four health facilities (Kibagabaga, Nyamata, Nyanza, and Ruhengeri) remained consistent in terms of staffing levels, number of deliveries and CD rate as outlined in the setting section (see line 119-145). However, we recognize that the economic impact of the COVID-19 pandemic, which disrupted livelihoods globally, could have influenced the ability of some individuals to access healthcare services in a timely manner. Unfortunately, our study did not collect data directly related to the socioeconomic status of participants or their financial challenges during this period, which limits our ability to directly assess this potential confounding factor. We have mentioned this as a limitation in the manuscript (line 364-370)

**B. RESPONSE TO MINOR ISSUES:**

**Response to comment 1.** We have revised the Methods section of the abstract to state that two non-parallel cohorts were compared. These changes can be found in the manuscript on lines 37–39.

**Response to comment 2:** We have removed the word "primary" and clarified the study’s objective for better clarity. These revisions can be found in the manuscript on lines 90–94

**Response to comment 3:** we would like to clarify that the health facilities included in the study are located in urban areas, but they function as referral centers for surrounding health centers situated in both urban and rural areas. We have updated the manuscript to reflect this information (see lines 115–117.

**Response to comment 4:** We have reviewed the formula and corrected the denominator, changing the “+” sign to a “-” sign, in accordance with the reference (Line 232)

**Response to comment 5:** Thank you for highlighting the importance of including additional information about the women in the study, such as education level and residence (urban/rural). Unfortunately, these variables were not collected because the data were extracted from antenatal care (ANC) registers, electronic medical records, delivery registers, and patients' files, and these sources do not contain information on education level or residency status (urban/rural).

We agree that the absence of these variables is a limitation of our study, as it restricts our ability to assess the potential influence of these factors on healthcare utilization and outcomes. This limitation has been acknowledged and discussed in the manuscript (see lines 361-364).

**Response to comment 6:**

- The title of Table 2 has been updated to "Incidence of Emergency CD by Women's Characteristics”.
- Table 2 has been revised to include the absolute counts, the proportion of emergency CD (row percentages), the 95% confidence intervals for the proportions, and the overall p-value, as suggested.
- Additionally, the same table has been generated separately for the pre-intervention and intervention periods. These tables have been included as supplementary materials to provide a more detailed comparison between the two periods.

**Response to Comment 7:** We have addressed this comment as follows:

- Weight at birth as an exposure: We acknowledge your observation that weight at birth is typically considered a newborn outcome. Based on your feedback, this variable has been removed from the analysis in Table 2.
- Uterine contraction at arrival: We agree that uterine contraction at arrival is part of the definition of the outcome. Therefore, this variable has been excluded from the adjusted logistic regression models, and Table 3 has been updated accordingly.
- Potential seasonality confounder: To address the potential influence of seasonality, we revised the facility characteristics described in the setting section to include the average monthly delivery rate for both the pre-intervention and intervention periods. (Starting line 125). The data showed that these averages were nearly identical across the two periods, suggesting no significant seasonal variation in the outcome. (line 121-145)

**Response to comment 8:** Upon review, we acknowledge that the mention of the stepwise method in the methodology section was a mistake. In fact, the stepwise procedure was not used in the selection of variables for the analysis. Given the limited number of variables in our dataset, there was no need to reduce the variable set further. Instead, we used bivariate and multivariable logistic regression models as described in the methodology section (line 204-208). We have revised the manuscript to remove any reference to the stepwise procedure.

**Response to comment 9:** As clarified in response to Comment 1, the study focuses on a single outcome: the rate of emergency cesarean deliveries, as stated in line 101 of the revised manuscript. Therefore, there is no need to build additional tables for other outcomes.

**C. Data sharing**

We have uploaded the dataset to Figshare, where it is publicly available for access and reference. You can view and download the dataset a <https://figshare.com/articles/dataset/Follow_up_of_pregnant_women_with_previous_cesarean_delivery_in_Rwanda_dataset_csv_csv/28341953?file=52116479>
